# Takotsubo Cardiomyopathy With an Unusually High Troponin Level Post-laparotomy for Small Bowel Obstruction Secondary to Adhesions in a Patient Presenting With Pelvic Inflammatory Disease

**DOI:** 10.7759/cureus.84685

**Published:** 2025-05-23

**Authors:** Abrar Z Khan, Upeshala A Jayawardena, David Luke

**Affiliations:** 1 Acute Medicine, University Hospitals of North Midlands NHS Trust, Stoke-on-Trent, GBR; 2 Surgery, University Hospitals of North Midlands NHS Trust, Stoke-on-Trent, GBR; 3 Colorectal Surgery, University Hospitals of North Midlands NHS Trust, Stoke-on-Trent, GBR

**Keywords:** high troponin, laparotomy, pid, small bowel obstruction, takotsubo cardiomyopathy (tcm)

## Abstract

Pelvic inflammatory disease (PID) triggering adhesion-related small bowel obstruction (SBO) is rare. The occurrence of Takotsubo cardiomyopathy (TCM) with an unusually high level of troponin I in the clinical course presents a unique diagnostic challenge. We report the case of a female patient in her 40s who had repeatedly presented to the hospital with abdominal pain and initially showed signs of PID, which later developed to SBO due to adhesions requiring laparotomy. Six days post-laparotomy, she experienced chest pain with ST-segment elevation on electrocardiogram (ECG) and high troponin I level at 16,804 ng/L (reference range: 0-39 ng/L), and bedside echocardiography showed apical ballooning with severely impaired left ventricular ejection fraction. Her cardiac biomarkers and echocardiogram features improved over the next few days making TCM the likely cause, with ST-segment elevation myocardial infarction (STEMI) being the main differential diagnosis. Besides the unique series of events that created the level of complexity, this case highlighted the importance of considering PID as a potential trigger for SBO, the cautious evaluation of troponin in acute coronary syndrome (ACS)-mimicking presentation, and, above all, the necessity of timely multidisciplinary team involvement in dealing with complex cases.

## Introduction

Pelvic inflammatory disease (PID) is a common condition affecting women of reproductive age and typically managed with antibiotics and supportive measures [1]. It can lead to complications such as infertility, chronic pelvic pain, ectopic pregnancy [2], and less commonly small bowel obstruction (SBO) [3]. 

SBO remains a common cause of mortality and morbidity that necessitates timely diagnosis and the early initiation of individualized management plan. Adhesions (65%), hernias (10%), Crohn's disease (5%), and neoplasm (15%) are all common aetiologies for SBO [4]. However, presentation with features attributable to PID that later develops into SBO presents a diagnostic dilemma and warrants a multidisciplinary team approach.

Takotsubo cardiomyopathy (TCM), also known as stress cardiomyopathy, apical ballooning syndrome (ABS), or broken heart syndrome, is now a well-recognized cause of acute coronary syndrome (ACS)-like presentations, typically occurring in post-menopausal women [5], without significant underlying atherosclerotic disease. Many different theories have been proposed to explain the pathophysiology of TCM; however, sympathetic stimulation with often identifiable emotional or physical triggers is widely reported [6]. Accounting for 2% of the cases presenting to the hospital with chest pain and ST-segment elevation [7], TCM is reported to cause a significantly lower level of troponin rise compared to ACS [5,8]. Apart from ST-segment elevation mostly in the anterior leads (56%), T-wave inversion is seen in 39% of the cases with TCM [9], while QT prolongation has also been reported [10]. The left ventricular dysfunction is usually transient, and treatment strategies focus on symptom control where the usage of diuretics, angiotensin-converting enzyme (ACE) inhibitors, and beta blockers has been described in existing literature [11].

We describe a case of TCM which had an interesting chain of events where multiple admissions with abdominal pain initially diagnosed as PID later on developed into adhesion-related SBO that required laparotomy. The patient went on to develop TCM with an unusually high troponin I level at 16,804 ng/L (reference range: 0-39 ng/L) six days after the surgery.

## Case presentation

**Figure 1:**
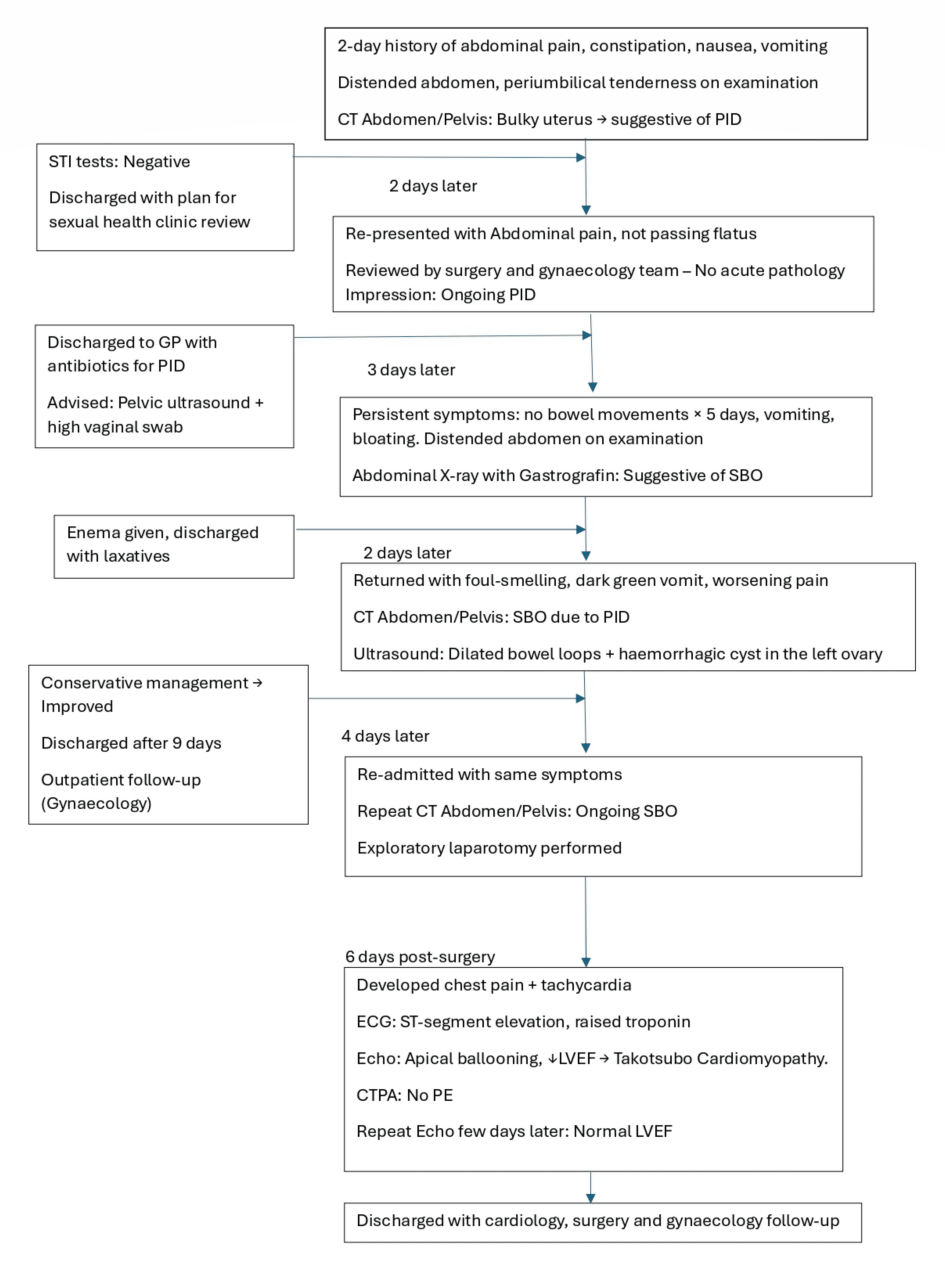
Flowchart depicting clinical events and progression since initial presentation STI: sexually transmitted infection; PID: pelvic inflammatory disease; GP: general practitioner; SBO: small bowel obstruction; ECG: electrocardiogram; LVEF: left ventricular ejection fraction; CTPA: computed tomography pulmonary angiogram; PE: pulmonary embolism

This patient had multiple presentations to the hospital, and a summary of the events is presented with a flowchart followed by a detailed description of the events (Figure 1).

A female patient in her 40s presented to the emergency department with a two-day history of abdominal pain and constipation along with nausea and vomiting. The patient was noted to have had bowel resection as a child and further laparotomy for adhesions when she was 30.

**Figure 2:**
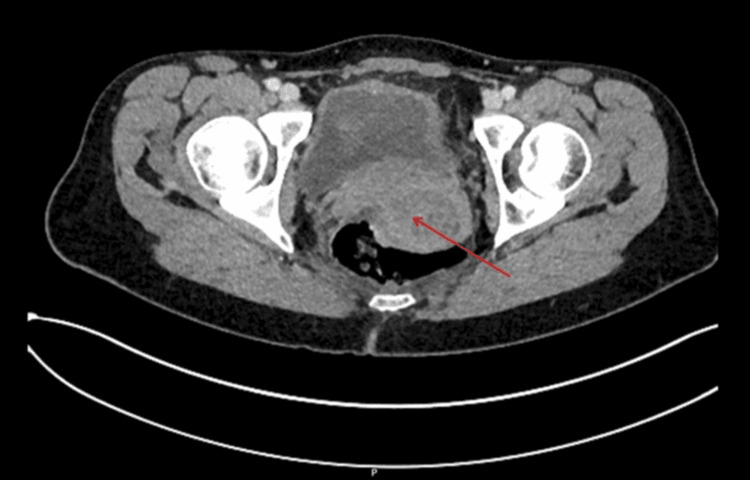
Computed tomography of the abdomen and pelvis showing bulky uterus (red arrow) with enlarged uterine cavity

On examination, her abdomen was distended with increased bowel sounds with tenderness over the periumbilical area. Blood tests showed a raised white blood cell count to 13.6×10,000/L and a mildly raised C-reactive protein (CRP) at 21. On the computed tomography of the abdomen and pelvis, a bulky uterus was noted in keeping with PID (Figure 2). She was recently tested for sexually transmitted infections (STI), noted to have negative results, and discharged with a sexual health clinic review.

**Figure 3:**
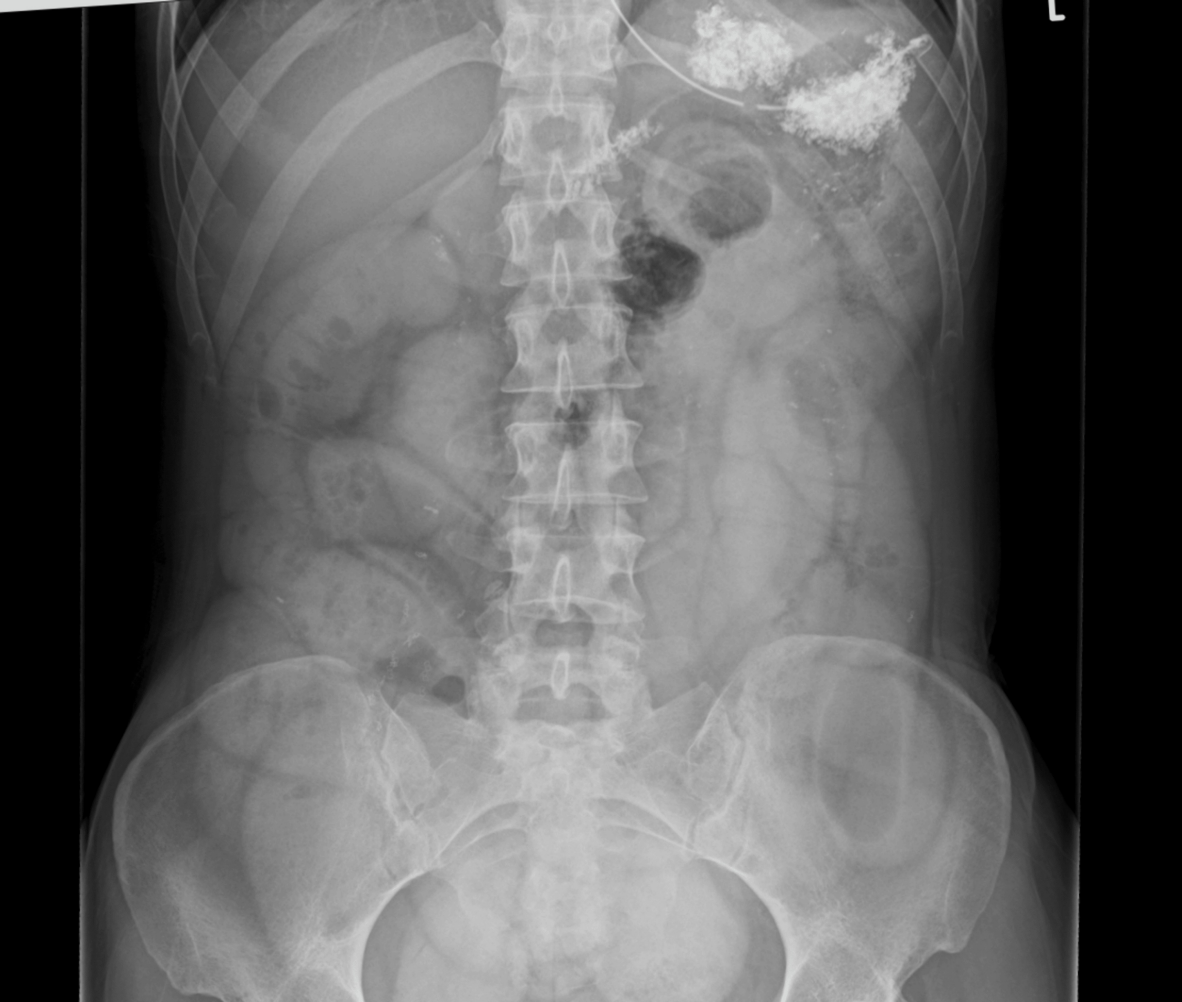
Abdominal X-ray showing diluted Gastrografin in dilated small bowel loops. It could not be confirmed if Gastrografin had passed to the colons

Two days later, the patient presented again to the hospital with complaints of the continuation of abdominal pain and not passing flatus. She was seen by general surgery and gynaecology who noted the previous computed tomography results and discharged the patient back to the general practitioner (GP) with antibiotics under the impression of continuing symptoms of PID. Further advice was provided to take a high vaginal swab at GP practice, and an outpatient pelvic ultrasound scan was booked. The patient presented again to the hospital after three days with complaints of the continuation of symptoms and having no bowel movements for five days. The pain during this admission was described as cramping in nature, where she felt bloated, and this was associated with several episodes of vomiting. Clinical examination showed that the abdomen was distended with no documented tenderness and no guarding or masses. An abdominal X-ray with Gastrografin was conducted during this admission which suggested ongoing SBO (Figure 3). Blood tests showed a CRP of 81, a white blood cell count of 10.8×10,000/L, and unremarkable urea and electrolytes. An enema was administered which, along with the Gastrografin, helped her to move her bowels, and the patient was discharged from the hospital with laxatives with a presumed diagnosis of SBO secondary to PID.

**Figure 4:**
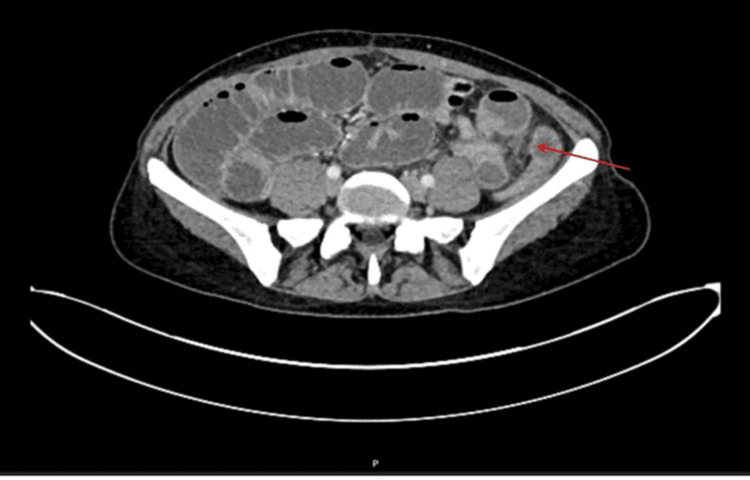
Computed tomography of the abdomen and pelvis showing dilated small bowel loops with transition point at distal ileus loops in the left iliac fossa (red arrow)

Two days later, the patient returned to the hospital with worsening symptoms, including persistent abdominal pain and vomiting of foul-smelling, dark green, partially digested food mixed with black lumps. She had not been eating and drinking well and had not opened bowels since her last bowel movement with enema during the previous admission despite regular macrogol administration at home. Another computed tomography of the abdomen and pelvis with contrast was organized that showed SBO, which was suggested to have been triggered by the PID (Figure 4).

**Figure 5:**
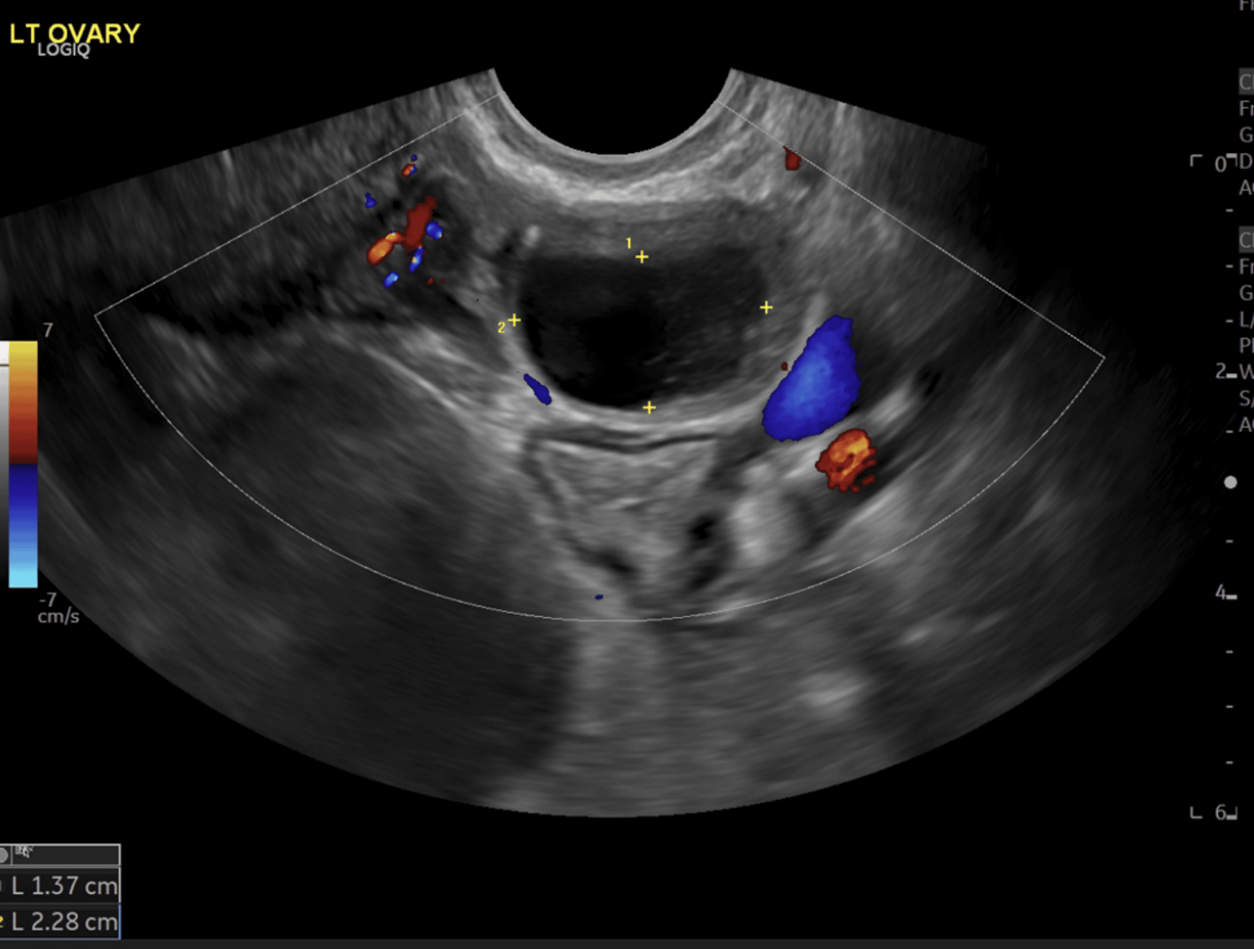
Transvaginal ultrasound with Doppler showing a haemorrhagic cyst in the left ovary

The patient was admitted under general surgery. Transvaginal and transabdominal ultrasound was conducted and noted to have dilated bowel loops which were in keeping with SBO and a haemorrhagic cyst in the left ovary (Figure 5). The patient was managed conservatively. Three days into the admission, the patient was able to open her bowels but passed loose stools. She was progressing well in herself with conservative management and was discharged after nine days with a gynaecology follow-up for the haemorrhagic cyst.

**Figure 6:**
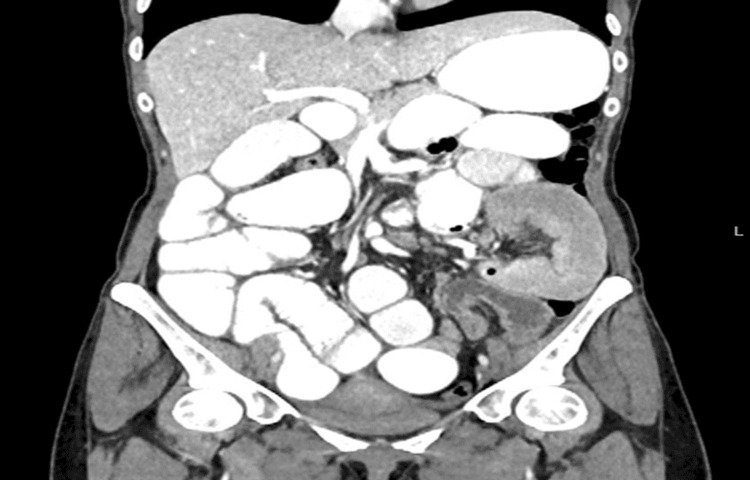
Computed tomography of the abdomen and pelvis with contrast showing dilated stomach and proximal small bowel loops with collapsed distal small bowel and colon

Four days following the discharge, the patient presented again to the hospital with the same symptoms as above and got admitted under the impression of SBO. Computed tomography of the abdomen and pelvis contrast was repeated which showed evidence of SBO possibly due to adhesions (Figure 6).

**Figure 7:**
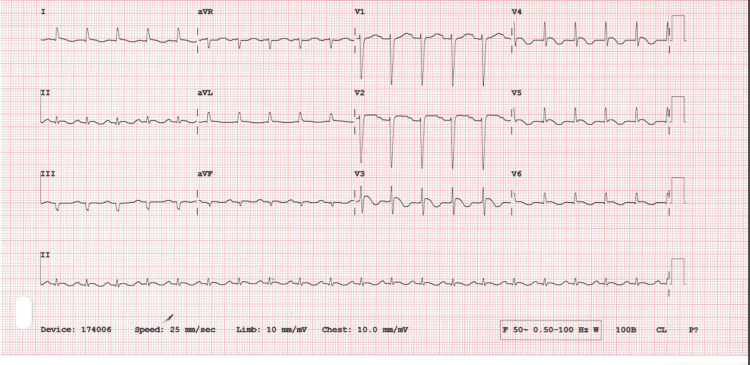
12-lead electrocardiogram showing ST-segment elevation in leads V2-V4 and T-wave inversion in leads V2-V6 aVR: augmented vector right; aVL: augmented vector left; aVF: augmented vector foot

The patient underwent exploratory laparotomy surgery to relieve the obstruction. Intraoperative findings revealed a significant burden of adhesions resulting in an ileocolic anastomosis. The point of obstruction was located in the small bowel, which was entangled in a knot of omentum, with proximal small bowel dilation up to the duodenojejunal junction. Distal to the obstruction, approximately 50 cm of collapsed small bowel was observed. Adhesiolysis was carried out, and a decision was made to resect the point of obstruction along with the formation of a new ileocolic anastomosis. Six days after the surgery, she complained of chest pain and was found to have persistent tachycardia with ischemic changes on the ECG and a very high troponin level (Figure 7).

**Figure 8:**
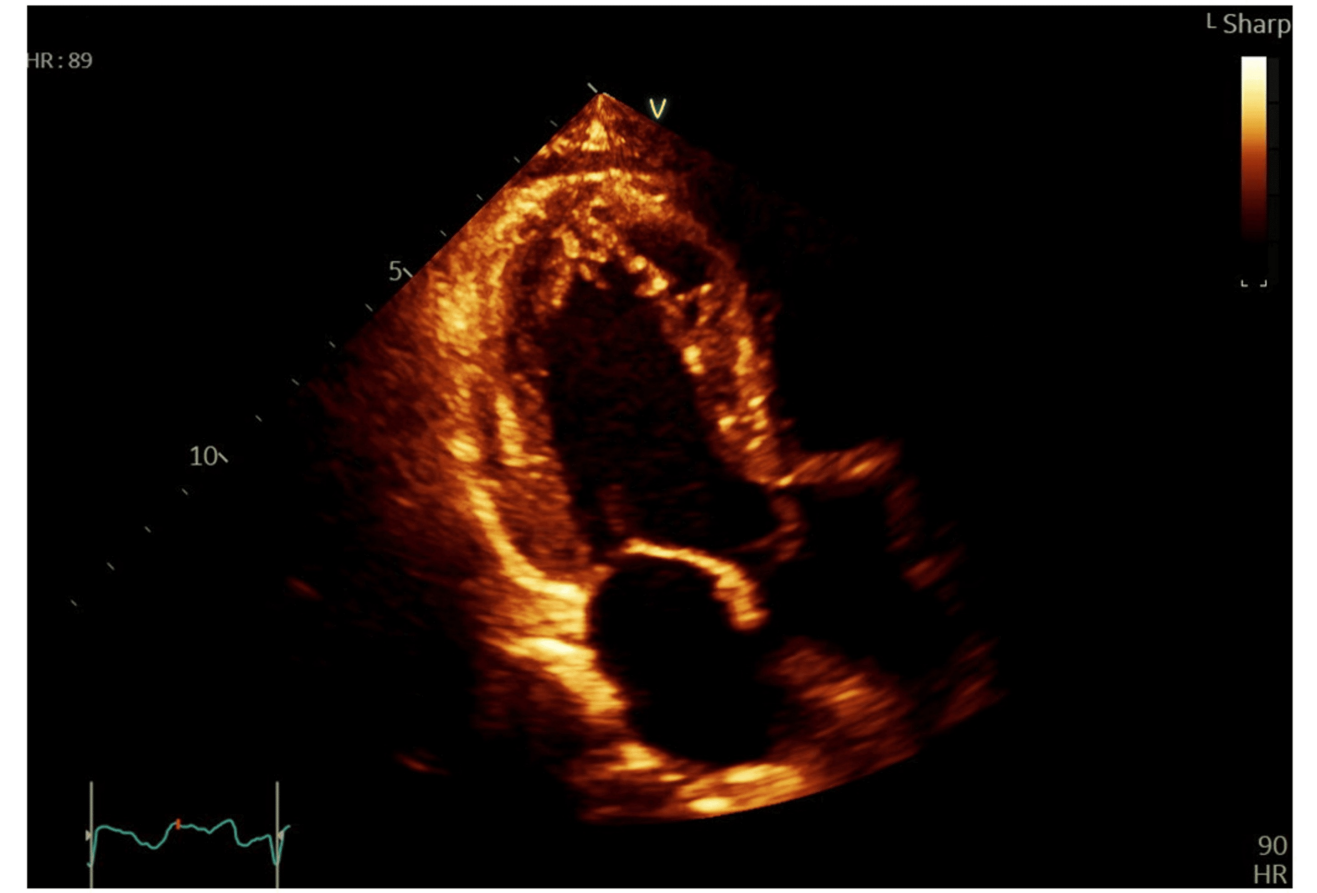
End-systolic apical view in transthoracic echocardiogram showing LV apical ballooning LV: left ventricular

Troponin I was unusually high at 16,804 ng/L (reference range: 0-39 ng/L). CRP was found at 127 and white blood cell count at 6.1×10,000/L prior to this event. Cardiology assessment was sought immediately, and transthoracic echocardiogram showed apical ballooning with severely impaired left ventricular ejection fraction (LVEF) (Figure 8).

Computed tomography pulmonary angiogram was negative for pulmonary embolism and did not show evidence of coronary artery disease. While ST-segment elevation myocardial infarction (STEMI) and TCM were the primary differentials, other potential causes such as pulmonary embolism, myocarditis, non-ischemic cardiomyopathy, and coronary artery spasm were also considered.

With the development of cardiac symptoms, the patient was treated with dalteparin 5000 units twice daily instead of starting aspirin 300 mg for suspected STEMI. Medication was optimized with bisoprolol 1.25 mg, candesartan 4 mg, and apixaban 5 mg on discharge. With typical features of TCM in echocardiogram and the rapid resolution of symptoms, the cardiology team decided not to undertake a coronary angiogram urgently.

Repeat echocardiogram done a few days after showed the complete resolution of left ventricular (LV) apical ballooning and a normal LVEF. Repeat troponin was 422 and CRP was noted coming down to 11. The patient was discharged with cardiology and surgical team outpatient appointments.

## Discussion

PID is known to cause adhesions even in previously unoperated abdomen but rarely can cause SBO when the inflammatory process extends further to the bowels. Although this patient had previous bowel surgery, initial presentation and imaging findings consistent with PID make definitive diagnosis challenging. While a relatively small number of cases of SBO due to adhesions caused by PID has been reported [3,12], the development of TCM six days after the abdominal surgery makes this a unique clinical encounter adding layers of complexity and diagnostic challenge.

Named after the Japanese word that means "octopus trap", TCM is a well-reported cause of ACS-mimicking presentation. Although described as being transient and reversible, TCM has comparable long-term outcome and mortality profile to that of ACS [13].

Intravenous injection of catecholamine and beta agonists giving rise to clinical features and different ballooning patterns of TCM [14] has emphasized the role of catecholamine-driven sympathetic activation in the pathophysiology of the condition, although different genetic [15] and hormonal factors have also been proposed [16]. Moreover, a meta-analysis by Cappelletti et al. looked at sepsis-associated TCM [17] that might link the underlying pathophysiological process to that of infection, particularly different biologic processes related to infection such as the release of inflammatory cytokines or endothelial dysfunction. The case we described could possibly have multiple pathophysiological processes leading to the development of TCM given the possible psychological stress of multiple admissions with troublesome abdominal pain, undergoing a major surgery, and the infectious process associated with complicated PID.

One of the interesting findings in this case is the very high level of troponins, whereas existing literature suggests that troponins greater than 26 times the upper limit of normal are more likely to have ACS [8]. Recent surgery, infection, and TCM itself could have played a role in causing a sharp rise in the cardiac biomarker; however, this presented a diagnostic challenge given typical features of apical ballooning seen on the bedside echo and the presence of definite physical stress. The quick resolution of the cardiac biomarker rise and reversal of the echocardiogram feature did establish the likely diagnosis to be TCM here, but it nevertheless highlighted the need for urgent careful evaluation from a specialist team. Early diagnosis helped in ruling out ACS which would have required urgent intervention with potential serious associated risk in this particular post-surgical case and helped in optimizing bleeding vs thromboembolic risk.

Some authors recommended classifying TCM as primary (psychic stress) and secondary where secondary is seen in already hospitalized patients [18]. Sadly, the secondary form of TCM is reported to show worse short- and long-term outcomes compared to primary in terms of mortality, recurrence, and readmissions [19] and hence becomes ever more important to be recognized in hospitalized patients such as in the case we described.

## Conclusions

This case underscores the importance of a multidisciplinary team approach when dealing with a complex presentation with diagnostic challenge and the timely collaboration among specialist teams such as gynaecology, surgery, and cardiology. Although the literature is still limited, PID may need to be acknowledged as a trigger for SBO regardless of whether the patient had previous abdominal surgery or not. Troponin elevation in TCM is usually mild; however, this case demonstrated an unusually high level (16,804 ng/L), suggesting that additional factors such as surgical stress or systemic inflammation may have contributed besides catecholaminergic drive alone. Further studies are required to better understand these different pathophysiological processes and to look at using different imaging modalities and biochemical parameters other than troponin to help develop more sensitive and specific diagnostic criteria to aid in differentiating TCM from ACS. Accurate diagnosis is of paramount importance as both TCM and ACS have similar mortality profiles; hence, the necessity of prompt assessment from the specialist team cannot be overstated.
